# Catamenial Epilepsy: Discovery of an Extrasynaptic Molecular Mechanism for Targeted Therapy

**DOI:** 10.3389/fncel.2016.00101

**Published:** 2016-04-22

**Authors:** Doodipala Samba Reddy

**Affiliations:** Department of Neuroscience and Experimental Therapeutics, Texas A&M University Health Science Center, College of MedicineBryan, TX, USA

**Keywords:** catamenial epilepsy, neurosteroid, GABA-A receptor, progesterone, mouse model

## Abstract

Catamenial epilepsy is a type of refractory epilepsy characterized by seizure clusters around perimenstrual or periovulatory period. The pathophysiology of catamenial epilepsy still remains unclear, yet there are few animal models to study this gender-specific disorder. The pathophysiology of perimenstrual catamenial epilepsy involves the withdrawal of the progesterone-derived GABAergic neurosteroids due to the decline in progesterone level at the time of menstruation. These manifestations can be faithfully reproduced in rodents by specific neuroendocrine manipulations. Since mice and rats, like humans, have ovarian cycles with circulating hormones, they appear to be suitable animal models for studies of perimenstrual seizures. Recently, we created specific experimental models to mimic perimenstrual seizures. Studies in rat and mouse models of catamenial epilepsy show enhanced susceptibility to seizures or increased seizure exacerbations following neurosteroid withdrawal. During such a seizure exacerbation period, there is a striking decrease in the anticonvulsant effect of commonly prescribed antiepileptics, such as benzodiazepines, but an increase in the anticonvulsant potency of exogenous neurosteroids. We discovered an extrasynaptic molecular mechanism of catamenial epilepsy. In essence, extrasynaptic δGABA-A receptors are upregulated during perimenstrual-like neuroendocrine milieu. Consequently, there is enhanced antiseizure efficacy of neurosteroids in catamenial models because δGABA-A receptors confer neurosteroid sensitivity and greater seizure protection. Molecular mechanisms such as these offer a strong rationale for the clinical development of a neurosteroid replacement therapy for catamenial epilepsy.

## Introduction

Catamenial epilepsy is a gender-specific epilepsy condition that is typified by the occurrence of seizure exacerbations in a cyclical pattern during particular phases of women’s menstrual cycles (Newmark and Penry, 1980; Reddy, 2004; Herzog et al., 2008). It appears that both partial and primary generalized epilepsies have been known to exhibit these cyclical fluctuations (Reddy, 2004). Since antiquity, it has been recognized that seizure susceptibility is greatly influenced by the menstrual cycle. In some cases, women diagnosed with an epileptic disorder that demonstrates changes in seizure frequency in a cyclical manner are routinely treated with antiepileptic drugs (AEDs) for which the seizures do not respond. In these cases, catamenial epilepsy is categorized as a specific form of pharmacoresistant epilepsy (Reddy and Rogawski, 2009). Most of these patients suffer from uncontrollable catamenial seizures, which could damage the brain and adversely impact their quality of life.

**FIGURE 1:**
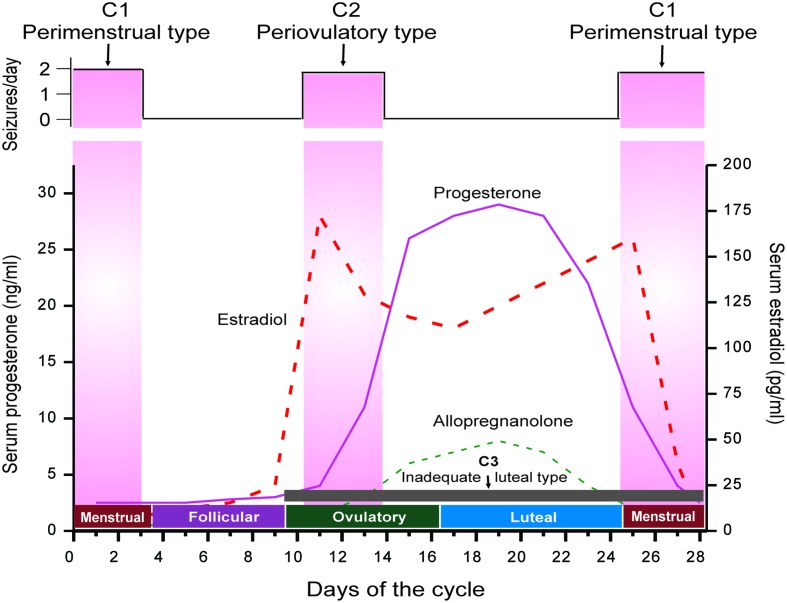
**The neuroendocrine basis of catamenial seizures during menstrual cycle.** The upper panel illustrates temporal occurrence of catamenial seizures and shows a strong relationship between seizure frequency and estradiol/progesterone levels. The lower panel illustrates the three types of catamenial epilepsy. The vertical pink bars (left and right) represent the likely period for the perimenstrual (C1) type, while the vertical pink bar (middle) represents the likely period for the periovulatory (C2) type. The horizontal dark gray bar (bottom) represents the inadequate luteal (C3) type, which typified by low level of progesterone and progesterone-derived neurosteroid allopregnanolone (AP) (*Adapted from* Reddy, 2014).

Catamenial seizure exacerbations affect 10–70% of women with epilepsy, many of these women being of reproductive age (Reddy, 2009). The large disparity in incidence is primarily due to differences in definition. Three types of catamenial seizures have been identified: Perimenstrual (C1), Periovulatory (C2), and Inadequate luteal-phase (C3) (Herzog et al., 1997; Reddy, 2014) (**Figure 1**). A specific pattern can be identified by charting menses and tallying seizures in a diary format, in addition to procuring a mid-luteal phase serum progesterone level to differentiate between normal and deficient luteal phases (Herzog et al., 2008; Quigg et al., 2009). The menstrual cycle is divided into four phases: (a) menstrual phase; (b) follicular phase; (c) ovulatory phase; and (d) luteal phase (**Figure 1**). The number of seizures is recorded, by phase, for a time period of no less than two menstrual cycles. Catamenial epilepsy can be diagnosed in women with both ovulatory and anovulatory cycles, in fact one study found that 16.5% of catamenial epilepsy patients had anovulatory cycles (Herzog et al., 2004). These women were found to show inadequate luteal phase (C3) or anovulatory luteal seizures which is the third type of catamenial epilepsy. By performing a complete review of menstruation patterns and seizure diaries along with the evaluation of serum progesterone levels, a diagnosis of catamenial epilepsy can be made because an increase in frequency of twofold or larger during one specific phase of the menstrual cycle is indicative of a problem (Reddy, 2009). The perimenstrual type is more common than other two types of catamenial epilepsy.

Experimental models have been developed that mimic perimenstrual seizures (i.e., catamenial epilepsy). This seizure condition can be induced either by electrical stimulation or with pharmacologic agents in rodents with suitable manipulation of neuroendocrine milieu (Reddy, 2009; Reddy et al., 2012). This review provides a brief overview of different experimental models of catamenial epilepsy for studies of new therapeutic strategies. It also describes our recent findings of an extrasynaptic molecular mechanism of catamenial epilepsy and its clinical potential for designing a rational therapeutic strategy. There are several features for an ideal catamenial epilepsy model (**Table 1**). An ideal animal model of epilepsy should reflect a pathophysiology akin to those of catamenial seizures in women with epilepsy. Human epilepsy is an intricate and complex brain disorder which encompasses a myriad of causes and seizure phenotypes, thus it is extremely doubtful that any single animal model can truly recapitulate the full spectrum of catamenial seizure features. Therefore, it is necessary to test the drug products and pathological mechanisms in a battery of animal models.

**Table 1:** Desirable features of animal model of catamenial epilepsy.

• Exhibit appropriate menstrual seizure phenotype
• Consistent with the neuroendocrine features of women with epilepsy
• Exhibit appropriate latency following steroid hormone fluctuation
• Show perimenstrual acute hyperexcitability and neuronal plasticity
• Express catamenial seizures following progesterone/neurosteroid withdrawal period
• Respond to drug therapy and exhibit resistance to certain anticonvulsants
• Allow rapid screening of novel compounds

## An Overview of Neuroendocrine Basis of Catamenial Epilepsy

Catamenial epilepsy is a multidimensional neurological disorder ascribed to a variety of origins (Reddy, 2013a–c). Epilepsy usually develops because of a genetic defect or after brain injury. Since there is no definitive evidence of genetic components, catamenial epilepsy is assumed to be an acquired disorder in many cases. A battery of mechanisms have been proposed as causes for catamenial epilepsy including: physiological variation in ovarian hormone secretion, fluctuations in water and electrolyte balance, and changes in AED levels (Scharfman and MacLusky, 2006; Reddy, 2009; Pack et al., 2011). The changes in the levels of steroid hormones circulating throughout the central nervous system are involved in the development of this epileptic condition (**Figure 1**). The role of estrogens in seizure susceptibility is complex; however, it is suggested that estrogen plays a role in facilitating some forms of catamenial seizures including periovulatory type (Logothetis et al., 1959; Bäckström, 1976; Bäckström et al., 1984; Bonuccelli et al., 1989; Edwards et al., 1999). The estrogens are widely recognized to cause proconvulsant effects (Reddy, 2009; Younus and Reddy, 2016). The periovulatory catamenial activities have been attributed to mid-cycle surges of estrogen which are generally unhindered by progesterone except in the early stages of the luteal phase.

Progesterone has powerful antiseizure effects in both animals and humans, and plays a critical role in the pathophysiology of catamenial epilepsy (Jacono and Robinson, 1987; Herzog, 1999; Reddy, 2009; Reddy and Jian, 2010). It has anticonvulsant activity in a variety of seizure models (Reddy et al., 2004). Natural cyclical fluctuations of progesterone during the menstrual cycle can influence catamenial seizure susceptibility in women with epilepsy (**Figure 1**). In this case, a decrease in seizures is seen during the mid-luteal phase, when serum progesterone levels are elevated, and an increase in seizures is seen during the premenstrual phase when progesterone levels fall. Changes in progesterone levels have been directly correlated with catamenial seizures. Progesterone is a key intermediate precursor for the synthesis of many neurosteroids in the brain. Neurosteroids are highly lipophilic steroid compounds that can rapidly modify the excitability of neurons through non-genomic mechanisms (Reddy, 2003). Numerous neurosteroids are shown to be biosynthesized within the brain (Agís-Balboa et al., 2006), the most widely studied being allopregnanolone (AP), allotetrahydrodeoxycorticosterone (THDOC), and androstanediol. In addition, because neuro steroids easily cross the blood–brain barrier, even if they are thus neurosteroids synthesized peripherally, they tend to build up in the brain (Mellon et al., 2001; Do Rego et al., 2009; Reddy, 2010).

The mechanism by which neurosteroids are able to rapidly alter neuronal excitability is through reversible interaction with synaptic and extrasynaptic GABA-A receptors. AP and similar neurosteroids are allosteric agonists that directly activate GABA-A receptors (Carver and Reddy, 2013). The mechanism of action is concentration dependent; at higher concentrations they directly activate the receptor whereas, at low concentrations neurosteroids potentiate GABA-gated currents. The GABA-A receptor consists of five subunits that form a chloride channel. So far, sixteen subunits have been identified (α1–6, β1–3, γ1–3, δ,𝜀,𝜃, and π subunits). Neurosteroids bind to distinct sites within the α- and β-subunits (Akk et al., 2005; Hosie et al., 2007; Chisari et al., 2010). The GABA-A receptor mediates both phasic inhibition (via synaptic) and tonic inhibition (via peri or extrasynaptic receptors) (Brickley and Mody, 2012). Neurosteroids can work on all receptor isoforms; however, they have a particular effect on the extrasynaptic δ-subunit GABA-A receptors which regulate tonic inhibition (Wohlfarth et al., 2002; Carver and Reddy, 2013; Carver et al., 2014). Thus, GABA-A receptors containing δ-subunits are greatly sensitive to neurosteroid potentiation. Tonic currents cause a shunting inhibition of neuronal networks thereby greatly reducing excitability. However, such extrasynaptic receptors are extremely insensitive to modulation by benzodiazepines (Reddy et al., 2015).

Neurosteroids are robust anticonvulsants that are efficacious for seizures induced by GABA-A receptor antagonists (Reddy, 2011). Neurosteroids have also been effective against pilocarpine-induced limbic seizures and kindling-induced seizures in animal models; however, neurosteroids have shown little effect or are only weakly active against seizures elicited by maximal electroshock (Kokate et al., 1994; Reddy and Rogawski, 2000a,b, 2002, 2010; Reddy, 2004, 2011). Additionally, neurosteroids have been highly effective in controlling seizures due to drug withdrawal, including ethanol and cocaine (Reddy and Woodward, 2004). In contrast to benzodiazepines, chronic exposure to neurosteroids does not lead to a tolerance of anticonvulsant activity (Reddy and Rogawski, 2000a). There is an increasing amount of evidence suggesting that endogenous neurosteroids play an important role in regulating epileptogenesis, in addition to the anticonvulsant activity of these compounds (Reddy et al., 2010; Reddy and Mohan, 2011).

In women diagnosed with epilepsy, it has been shown that both endogenous neurosteroids and plasticity in GABA-A receptor subunits may be factors in perimenstrual catamenial seizure susceptibility (Reddy, 2014). Seizure control can be lost when neurosteroid levels fluctuate (Herzog and Frye, 2003). Even in women with a normal menstrual cycle, neurosteroids have been associated in perimenstrual seizure exacerbations. This has led to some hypotheses that the withdrawal of progesterone-derived neurosteroids leads to enhanced excitability (Smith et al., 2007). Strong evidence has been reported that shows ovarian cycle-linked fluctuations in neurosteroids modulate GABA-A receptor plasticity. We reported that neurosteroid withdrawal (NSW) upregulates the expression of the α4 and δ-subunits in the hippocampus (Gangisetty and Reddy, 2010; Wu et al., 2013; Carver et al., 2014). Such subunit plasticity is also noted in other withdrawal models (Smith et al., 1998b; Gulinello et al., 2001; Maguire et al., 2005; Maguire and Mody, 2007).

## Molecular Insights On Neurosteroid Withdrawal

Neuronal GABA-A receptors undergo cyclical variations in women. GABA-A receptor subunit expression is not static either, but undergoes alterations which compensate for fluctuations and changes in endogenous hormonal levels and exogenously administered agents which modulate GABA-A receptors, such as neurosteroids or benzodiazepines. Extended exposure to AP in rats causes upregulation of the α4-subunit in hippocampus resulting in a decreased sensitivity to benzodiazepine (Smith et al., 1998a,b; Gangisetty and Reddy, 2010). The α4 subunit preferentially co-assembles with δ, rather than γ2, to form extrasynaptic GABA-A receptors. Increases in the expression of the δ-subunit within the hippocampus can be seen as a result of AP administration. Additionally, the tolerance of benzodiazepines can increase at these tonic currents, creating a heightened insensitivity to these compounds. Higher δ-subunit expression has been reported after exposure to progesterone, presumably caused by the conversion of progesterone to AP.

The exact link between α4-subunit plasticity and excitability is unclear. The normally low prevalence of the α4-subunit into synaptic GABA-A receptors generates synaptic currents with accelerated decay kinetics, causing an overall decrease in charge transfer, which likely results in reduced inhibition. Neurosteroids that modulate GABA-A receptors produce a prolongation of the decay of GABA-mediated synaptic currents. Hence, during the luteal phase when there are elevated levels of AP, the acceleration due to α4-substitution is balanced. However, when the levels fall during menstruation, the synaptic inhibition is greatly reduced, causing heightened excitability that predisposes one to seizures, along with other effects.

Upregulation of α4 has been linked to a compensatory reduction in α1 expression. Some research has suggested that removal of neurosteroids is related to a downregulation in α1 and γ2 subunit expression (see Reddy and Rogawski, 2009). It is known that the γ2 subunit is necessary for synaptic clustering and targeting of GABA-A receptors in dendrites. Heightened excitability might be the outcome of a net reduction of synaptic GABA-A receptors. It is important to note that if exposure to neurosteroids exceeds 72 h, then there is a return of α4-subunit expression to normal levels, indicating that the rise in α4-subunit expression is temporary. However, if there are extended periods of neurosteroid exposure, then withdrawal causes a powerful rebound rise in α4-subunit expression. It is the *withdrawal* of neurosteroids near the beginning of menstruation, instead of the extended exposure to neurosteroids during the luteal phase that is probably to be most pertinent to the enhanced excitability and greater seizure susceptibility in perimenstrual catamenial epilepsy (Smith et al., 2007; Carver et al., 2014). Chronic exposure to neurosteroids is correlated by the downregulation of δ-subunit expression and extrasynaptic GABA-A receptors. This alteration is suspected to be a compensatory mechanism, to avoid extreme sedation resulting from elevated neurosteroid levels acting on sensitive δ-containing GABA-A receptors (Maguire and Mody, 2007). When there is a withdrawal of neurosteroids, δ-expression quickly recovers and there is striking upregulation following perimenstrual-like condition (Carver et al., 2014). When recovery does not occur quickly enough, there is the possibility of an enhancement of excitability because of a reduction in tonic inhibition mediated by extrasynaptic GABA-A receptors in the comparative absence of neurosteroids.

## Current Animal Models of Catamenial Epilepsy

Animal models of epilepsy continue to play an important role to better understand the pathophysiology and discovery of new AEDs. Traditional seizure models were established by acutely inducing seizures in naïve animals. These models are not appropriate for catamenial epilepsy because they do not provide the correct avenue to test specific therapies targeted to the disorder. The models of catamenial epilepsy are clearly different from induction models utilizing pilocarpine, chronic kainic acid, or hippocampal kindling because they create extreme damage and a remodeling response in the brain, thus resulting in recurrent seizures. Generally, catamenial epilepsy models should be characterized to specifically mimic alterations in seizure vulnerability caused by menstruation and ovarian hormone fluctuations (Reddy, 2009). During the luteal phase of menstruation in humans, levels of circulating progesterone are elevated for 10–12 days prior to declining. These phenomena can be faithfully reproduced in rodents with specific neuroendocrine manipulations. Because mice and rats, like humans, have ovarian cycle with circulating hormones, they appear to be suitable animal models for studies of perimenstrual seizures.

Three broad categories of models have been described in animals that partially simulate the pattern of catamenial seizures (Reddy, 2009). For the first category, study protocols focus on the luteal phase. The mimicry is created by the induction of prolonged elevated levels of progesterone and estrogens then creating a sudden fall to imitate menstruation in rodents. These categories of models have been named: (a) pseudopregnancy, (b) chronic progesterone, and (c) progesterone (neurosteroid) withdrawal (Moran and Smith, 1998; Smith et al., 1998a; Reddy et al., 2001). The second category is built on the concept of the normal estrous cycle; sometimes this model requires administrating exogenous hormones that replicate the stages of the estrous cycle in ovariectomized rodents (Frye and Bayon, 1998; Wu et al., 2013). These functional models are closer to recreating the typical ovarian cycle and are therefore more comparable models. Finally, the third category consists of utilizing epileptic animals and exposing them to both steroid hormones and NSW. The severity and frequency of spontaneous seizures are the primary endpoints to evaluate the exacerbation of catamenial-like seizure (Reddy and Zeng, 2007; Reddy, 2013a).

### Rat Models

There are rat models of catamenial epilepsy which administer progesterone by using techniques that range from silastic implants to administering several daily injections (Costa et al., 1995; Moran and Smith, 1998; Smith et al., 1998a). These models simulate the elevated progesterone levels present during luteal phase. Withdrawal of either the progesterone containing implants or termination of chronic progesterone treatment creates a sudden decrease in progesterone (neurosteroids) levels that could mimic the hormonal milieu of menstruation. The withdrawal of progesterone has been linked to the marked decrease in seizure threshold, similar to finasteride-induced NSW (Moran and Smith, 1998; Smith et al., 1998a). The current rat models are outlined briefly in **Box 1**.

**Box 1. Rat catamenial epilepsy models.**

**FIGURE 2:**
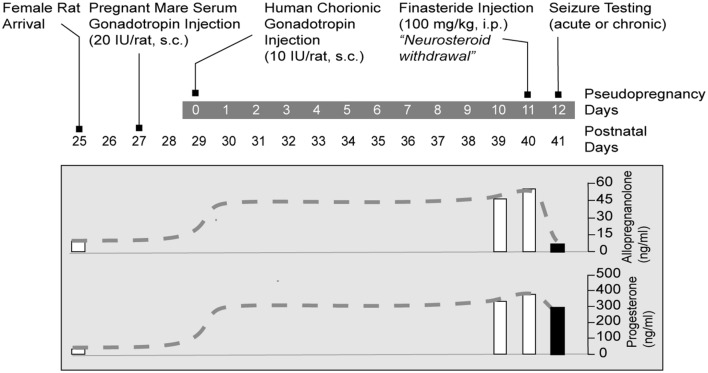
**Gonadotropin protocol for rat NSW model of catamenial epilepsy.** Rats are treated sequentially with gonadotropins for inducing prolonged elevated serum progesterone levels. On day 11, animals are treated with finasteride, which blocks the conversion of progesterone to the neurosteroid AP, resulting in “neurosteroid withdrawal.” Acute seizure testing is performed on the day following finasteride administration. For studies of spontaneous seizures, the gonadotropin treatment was initiated 5 months after pilocarpine-induced epilepsy. Progesterone and AP plasma levels are represented as dashed lines schematically illustrate time course of the fluctuations in hormone levels (*Adapted from* Reddy and Rogawski, 2009).

**FIGURE 3:**
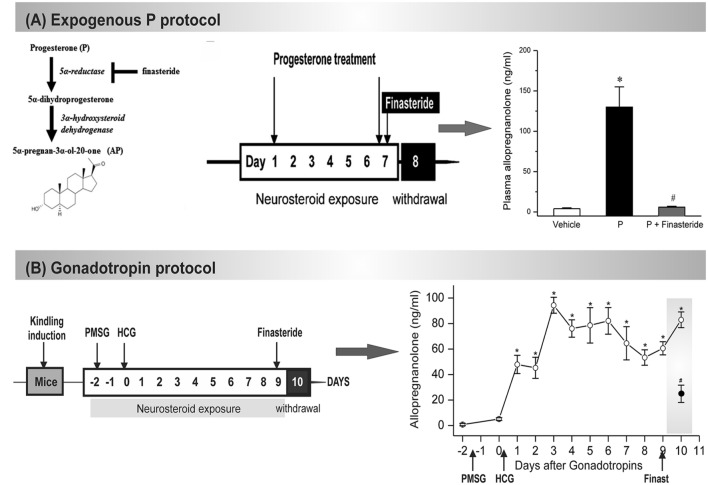
**Mouse NSW models of catamenial epilepsy. (A)** In the exogenous protocol, progesterone was administered twice daily for 7 d, and finasteride was administered on the final day, resulting in NSW. Data collections were done 24 h after induction of withdrawal (day 8). The resulting plasma levels of AP were estimated at day 8 in mice following treatment with vehicle (β-cyclodextrin solution), progesterone (P), and P + finasteride (NSW). Each bar represents the mean ± SEM (*n* = 6 mice per group). *p < 0.01 vs. vehicle; #*p* < 0.01 vs. P-treatment group (*Adapted from* Carver et al., 2014). **(B)** In the gonadotropin-based protocol, fully kindled mice were treated with pregnant mare’s serum gonadotropin (PMSG, 5 IU, s.c.) at 3 PM followed 48 h later human chorionic gonadotropin (HCG, 5 IU, s.c.) at 1 PM. Then, on day 9 they were given finasteride (50 mg/kg, i.p.). Plasma AP levels were estimated in mice following treatment with gonadotropins and finasteride (50 mg/kg, i.p.) paradigm for induction of neurosteroid withdrawal (NSW). The dramatic decline in neurosteroid levels 24 h after finasteride would create a state of NSW (*Adapted from* Reddy et al., 2012).

**(1) The pseudopregnancy model**. ***Approach*:** It was the first model of perimenstrual catamenial epilepsy utilizing the concept of pseudopregnancy (Reddy et al., 2001). In this model, a state of persistently elevated progesterone in female rats was induced by pseudopregnancy and the augmented neurosteroid levels including AP were abruptly withdrawn by blocking its synthesis using the 5α-reductase inhibitor finasteride. It was based on the hypothesis that increased susceptibility of catamenial seizure is caused by an abrupt decline (“withdrawal”) in circulating levels of progesterone and, consequently, of levels of its metabolite AP in the brain. ***Method*:** In this model, a persistently elevated level of progesterone in young female rats was induced by a standard gonadotropin regimen (**Figure 3A**). On day 11, animals are treated with finasteride to trigger “neurosteroid withdrawal.” Acute seizure testing was performed on the day following finasteride (day 12). The drop in AP levels following finasteride treatment was significant (**Figure 2**). Acute withdrawal of AP resulted in an increase in seizure susceptibility to pentylenetetrazol, but long-term treatment with finasteride did not, mimicking the situation in catamenial epilepsy. In another model, withdrawal of progesterone (neurosteroid) was obtained by ovariectomy (Moran and Smith, 1998). This model was associated with decreased seizure threshold to picrotoxin and reduced sensitivity to benzodiazepines. The progesterone withdrawal-induced hyperexcitability fits well an increase in seizure susceptibility in rodents (Voiculescu et al., 1994; Frye and Bayon, 1998) and also with clinical perimenstrual seizure patterns (Herzog et al., 1997).

**(2) The exogenous progesterone model. *Approach*:** This model is based on direct progesterone administration for extended periods using silastic implants or multiple daily injections, and then cessation of progesterone for inducing withdrawal (Smith et al., 1998a). ***Method*:** There are many methods in which steroid progesterone was administered for extended periods including silastic implants or multiple daily injections (Costa et al., 1995; Moran and Smith, 1998; Smith et al., 1998a). These models mimic the high P levels found during luteal phase of the menstrual cycle. Withdrawal of progesterone containing implants or cessation of chronic progesterone treatment induces an abrupt decline of progesterone levels that could simulate the hormonal milieu of menstruation. Akin to finasteride-induced neurosteroid withdrawal, withdrawal of progesterone has been associated with a marked decrease in seizure threshold (Moran and Smith, 1998; Smith et al., 1998a). A similar predisposition to seizures is observed upon abrupt discontinuation of benzodiazepines (File, 1990) and ethanol (Kokka et al., 1993; Reilly et al., 2000).

**(3) The spontaneous seizure model. *Approach*:** Unlike previous models that have utilized normal or healthy animals to study neurosteroid withdrawal, this model was created using chronic epileptic rats to study the impact of neurosteroid withdrawal on spontaneous perimenstrual-like seizures (Reddy and Zeng, 2007). It was devised again based on the hypothesis that those cyclic episodes of withdrawal would exacerbate seizure occurrence in rats with preexisting epilepsy. ***Method*:** The pilocarpine model was used for inducing chronic epilepsy with spontaneous recurrent seizures. Due to certain reproductive impact of other chronic epilepsy models, a modified lithium-pilocarpine model was utilized to create a stable epileptic condition with minimal impact on reproductive function (Reddy and Zeng, 2007). Rats with stable spontaneous seizures were then subjected to repeated pseudopregnancy-finasteride paradigm to model menstruation-like neurosteroid exposure and withdrawal. Neurosteroid withdrawal was associated with a significant ‘catamenial-like’ exacerbation of spontaneous seizures in epilepsy rats. The withdrawal period represents a crucial intervention phase for testing of potential treatments, and therefore, this chronic model may be useful for testing novel therapies for the perimenstrual catamenial epilepsy.

In order to improve understanding of the neurobiology of perimenstrual epilepsy and to investigate possible therapeutics, we characterized a rodent model designed to imitate the hormonal changes thought to be involved with perimenstrual catamenial epilepsy (Reddy et al., 2001). Rodents are the main mammalian species used in experimental research and have an estrous cycle that lasts 4–5 days. Because the human menstrual cycle is 28 days, research based on the fluctuations in seizure susceptibility during the rodents’ normal estrous cycle do not result in accurate or relevant data predicted of human disease (Finn and Gee, 1994). So, the model we present simulates human hormonal menstrual changes within the rat. The main criteria for this model is to elevate and prolong the levels of progesterone and then follow this period of high progesterone with the removal of hormone to stimulate the reduction in ovarian secretions which occur during menstruation. A sudden cessation of progesterone after chronic administration for 3 weeks leads to proconvulsant effects (Smith et al., 1998a,b). This published protocol is a very basic model of catamenial epilepsy. Advancing the model is accomplished by creating “pseudopregnancy” which is more physiologically relevant. This is done by inducing increased progesterone levels (and mimicking other changes of hormone levels in the human luteal phase such as elevated estrogen) by administering human chorionic gonadotropin and pregnant mare’s serum gonadotropin at the same time (**Figure 2**). Pseudopregnancy in the rat can last 12–13 days, during which estrogen levels are similar to those in the luteal phase of the human. Sudden withdrawal of progesterone-derived neurosteroids can be accomplished by administration of a 5α-reductase inhibitor which blocks neurosteroid synthesis or by performing an ovariectomy (Kokate et al., 1999; Reddy and Ramanathan, 2012). NSW is linked with enhanced susceptibility to seizures (Smith et al., 1998a,b; Reddy et al., 2001). We suggest that the phase of increased seizure susceptibility characterizes a model of human perimenstrual catamenial seizure exacerbations.

The “neurosteroid withdrawal (NSW)” model had previously only been used in rats that were otherwise normal and healthy; seizures were induced with pentylenetetrazol. Recently, this model has been used with female rats with a history of status epilepticus induced by pilocarpine and ultimately displayed recurrent seizures spontaneously (Reddy and Zeng, 2007). As demonstrated in the study, these rats showed a transitory rise in the occurrence of spontaneous seizures following NSW (Reddy, 2009). Utilizing animals that were epileptic and spontaneously displaying recurrent seizures, has better relevancy and authenticity for catamenial epilepsy than those with acute seizure threshold outcomes. The period of withdrawal represents a crucial intervention phase for the testing of potential therapeutics. While this approach is possibly more applicable to human epilepsy, it is considerably more demanding of personnel and less suited for comprehensive pharmacological investigations.

### Mouse Models

Although rat models are extremely useful for testing new therapeutic approaches for catamenial epilepsy (Reddy and Rogawski, 2001), there are also a few mouse models that recapitulate the neuroendocrine and clinical features of catamenial epilepsy. Mouse models of catamenial epilepsy are essential for molecular and genetic investigations. The current mouse models are outlined briefly in **Box 2**. Recently, we developed two distinct mouse models of perimenstrual catamenial epilepsy (Gangisetty and Reddy, 2010; Reddy et al., 2012) (**Figure 3**). These models are based on the rationale that a decrease in seizure susceptibility occurs when neurosteroid levels are elevated (luteal phase) and an increase in seizure susceptibility occurs during the withdrawal period (perimenstrual periods), which is associated with plasticity changes of the GABA-A receptor subunits. First, a chronic and recurring seizure condition is generated using the hippocampus kindling model in female mice. Second, the fully kindled mice are subjected to fluctuating levels of neurosteroids, like in the ovarian cycle. Here, we utilized two distinct pharmacological approaches to induce elevated neurosteroid levels: (a) chronic exogenous progesterone treatment protocol, and (b) gonadotropin regimen for induction of endogenous synthesis. In the first series, mice were treated with progesterone for 7 days. On the morning of the 7th day, mice were administered the finasteride to NSW to model perimenstrual seizures (**Figure 3**) (Gangisetty and Reddy, 2010).

**Box 2. Mouse catamenial epilepsy models.**

**(1) The exogenous progesterone model. *Approach*:** In this model, mice received chronic injections of progesterone and the resulting elevated neurosteroid AP was abruptly withdrawn by blocking its synthesis from progesterone by administration of finasteride (Omkaram and Reddy, 2010; Carver et al., 2014). ***Method*:** A state of neurosteroid withdrawal was induced in mice by an exogenous progesterone treatment protocol (**Figure 3A**). To produce prolonged elevated levels of neurosteroids that more closely model the luteal changes in women, mice were treated with progesterone (25 mg/kg s.c.), twice daily at 9 AM and 6 PM for 7 days. On the morning of the 7th day, mice were injected with finasteride (50 mg/kg i.p.). Animals were examined 24 h after finasteride (neurosteroid withdrawal, day 8). Progesterone was administered rather than the neurosteroid AP because it is known that elevated circulating levels of progesterone, such as those found during the luteal phase, are readily converted to neurosteroids in the brain regions that express neurosteroid synthesizing enzymes (**Figure 3A**). The progesterone administration protocol results in a high physiological concentration of AP in plasma, and an acute withdrawal was evident by a nearly complete decline in AP within 24 h after finasteride (**Figure 3A**). This neuroendocrine manipulation was implemented in fully-kindled mice. Mice were stimulated until they had exhibited stage 5 seizures and then they were subjected to neurosteroid withdrawal. Changes in seizure activity during neurosteroid withdrawal in mice in the hippocampus kindling model. There was a decreased threshold to induce generalized seizures, increased seizure duration, and time-course of the percent of animals exhibiting generalized seizures (stage 4/5) at 50% of regular ADT current. This augmented seizure susceptibility is an index of perimenstrual-like seizure exacerbation.

**(2) The gonadotropin model**. ***Approach*:** In this model, fully-kindled mice exhibiting a stable stage 5 seizures were utilized to better simulate a perimenstrual-like seizure condition (Reddy et al., 2012). Mice received a regimen of gonadotropins to elevate the endogenous neurosteroids, and then were drastically reduced with the administration of finasteride. It is based on the premise that steady physiological levels of elevated neurosteroid more closely resemble that of the neuroendocrine milieu of perimenstrual period. ***Method*:** A state of elevated neurosteroid was induced in female mice by a sequential gonadotropin regimen (Brooke et al., 2007). To produce prolonged elevated levels of progesterone and neurosteroids that more closely model the luteal changes in women, mice were treated with pregnant mare’s serum gonadotropin (5 IU, s.c.) at 3 PM followed 48 h later by human chorionic gonadotropin (5 IU, s.c.) at 1 PM (**Figure 3B**). The day of the second gonadotropin injection was considered day 1 of elevated neurosteroids. On the morning of the 9th day, mice were injected with finasteride (50 mg/kg, i.p.) to produce an abrupt decline in neurosteroid levels to more closely model perimenstrual changes in women. Animals were tested 24 h after finasteride administration (neurosteroid withdrawal). The gonadotropin administration protocol resulted in high circulating levels of AP, and an acute withdrawal was evident by a significant decline in AP 24 h after finasteride administration (**Figure 3B**). Elevated neurosteroid exposure reduced seizure expression in fully kindled mice. Fully-kindled mice subjected to neurosteroid withdrawal showed increased generalized seizure frequency and intensity and enhanced seizure susceptibility. They also showed reduced benzodiazepine sensitivity and enhanced neurosteroid potency, similar to the clinical catamenial seizure phenotype.

In the second series, neurosteroid levels were elevated by way of sequential gonadotropin treatment, which was followed by finasteride treatment to produce a withdrawal state (**Figure 3**) (Reddy et al., 2012). In both approaches, elevated neurosteroid exposure reduced seizure expression and susceptibility in fully kindled mice, indicating the protection offered by neurosteroids. Mice that were fully kindled and exposed to NSW presented with enhanced seizure susceptibility, elevated intensity and increased frequency. In pharmacological studies, these mice also showed enhanced neurosteroid potency and reduced sensitivity to benzodiazepine, akin to the clinical phenotype. Changes in seizure susceptibility and responses to antiseizure drugs are linked to an upregulation of α4- and δ-subunits of hippocampal GABA-A receptors (Gangisetty and Reddy, 2010; Reddy et al., 2012). The gonadotropin paradigm appears more physiologically similar than the exogenous pharmacological progesterone treatment. These mouse models of perimenstrual catamenial epilepsy can be used in the investigation of disease pathology and for the development of new treatment strategies.

These protocols are similar and consistent to the pseudo pregnancy model (Reddy and Rogawski, 2001; Reddy et al., 2001) as well as typical progesterone approaches used historically for the induction of NSW (Moran and Smith, 1998; Moran et al., 1998; Smith et al., 1998a,b). Although it is impractical to expect perfect replication of the endocrine environment of the human cycle in mouse models, these are physiologically similar to the perimenstrual phase. The gonadectomy model was not used due to likely difficulty of interpretation associated with total depletion of ovarian-derived hormones. Furthermore, the mice would require hormone replacement after such a procedure, which could have varying effects on seizures dependent on the age, hormone dose, and duration of treatment (Scharfman et al., 2005; Shen et al., 2010).

## Relevancy and Validation of Experimental Models

There are many advantages to using rodents, particularly mice and rats, as models in epilepsy research. The anatomic and physiologic similarities between humans and rodents make the small rodents the most widely used animal model for a variety of seizure disorders. Most studies of acute seizures and chronic epilepsy induced by chemoconvulsants have been carried out in rats and mice. The short generation time, relatively small size, and accelerated life span of rats and mice help maintain efficient space and time periods, which allows performance of a manageable study. Furthermore, most behavioral seizure scoring systems have been developed and validated in rats and mice; hundreds of transgenic mice are available, allowing researchers to investigate how specific genes function in epilepsy. Specifically as it relates to the field of perimenstrual seizures, the sensitivity of rats and mice to different anticonvulsants closely resembles that of humans. For example, the lower efficacy of diazepam and valproate to protect against perimenstrual seizures in rat models is similar to clinical reports (Reddy and Rogawski, 2000b).

In order for animal models of catamenial epilepsy to be validated, they are required to display certain criteria and be representative of the human condition (Reddy, 2009). Furthermore, the models should have the ability to elicit a similar epilepsy-like state as well as a pathophysiology that mirrors the disease in humans (**Table 1**). The NSW model meets many of these criteria and certainly offers advantages over the use of conventional seizure models (Reddy, 2009). The models of catamenial epilepsy previously described are based on similar physiological dynamics of ovarian progesterone secretion during the menstrual cycle, which cannot be simulated in the exogenous drug delivery models. In pseudopregnancy, secretion of progesterone by the luteinized ovaries occurs in a physiologically appropriate episodic fashion and leads to plasma progesterone levels that are within the normal physiologic range. Additionally, the 9 days elevation of progesterone and AP levels seen in the rodent model closely matches the 10 days increase in AP levels during the luteal phase of humans. Even though the exact mechanisms and etiology of catamenial epilepsy are not wholly understood, the NSW model simulates the AP-to-estrogen ratio changes that are believed to be critical in perimenstrual catamenial epilepsy. Female rats with spontaneous recurrent seizures are more appropriate to model catamenial epilepsy. There are some difficulties in this, such as controlling a consistent induction of epileptogenesis in female rats as well as potential abnormities in maintaining regular estrous cycles (Amado and Cavalheiro, 1998). Moreover, it is important to note that the actual endocrine conditions that exist in the human menstrual cycle are different from those observed in animal models of catamenial epilepsy; the estrous cycle duration in most rodents is approximately 4–7 days, and the menstrual cycle in women lasts about 28 days.

Catamenial seizures are often seen in women diagnosed with epilepsy; therefore, research models should also utilize animal with preexisting epilepsy. “Kindling” is an appropriate background paradigm for characterizing a perimenstrual catamenial epilepsy model. Hippocampus kindling is commonly used as a model of human complex partial seizures. In contrast to pilocarpine-induced epilepsy, the kindling model will not result in neuronal loss and preserves the reproductive capability in the rodent. Using the NSW paradigm, we developed a mouse kindling model and successfully utilized it to test new drugs/therapeutics that prevent kindled catamenial seizures (Reddy et al., 2012). The gonadotropin-induced endocrine state is more physiologically similar to the hormonal milieu of the menstrual cycle than the paradigms that employ exogenous progesterone or AP treatment (Shen et al., 2005; Gangisetty and Reddy, 2010). It is hypothesized that local production of neurosteroids happens in areas of the brain like the hippocampus and amygdala because these are germane to epilepsy (Reddy, 2013c). Seizure exacerbation observed in these models is due to the reduction of neurosteroids in the brain. Consequently, NSW associated with increased exacerbation of seizures might signify a transitory surrogate marker for perimenstrual catamenial epilepsy.

Non-rodent species, particularly canines, hold a unique advantage over rodents because epilepsy is a natural disease in some dogs (Patterson, 2014; Packer and Volk, 2015). In fact, epilepsy is the most common neurologic disease in dogs and is considered to have a genetic basis in many of its forms (Ekenstedt and Oberbauer, 2013). Undoubtedly, canines have a number of advantages. Since the 1970s dogs have been used as a limited comparative model, particularly in the areas of drug therapy, dietary therapy, and other therapeutic devices for epilepsy. The idiopathic epilepsy appears similar between humans and canines; therefore dogs represent the most appropriate animal model of epilepsy. However, canines may not be the best model for catamenial epilepsy. Striking ovarian cycling differences exist in dogs, including variable or long interestrus period, making it difficult to study catamenial seizures in female dogs (Reddy and Gadsby, 2009). Pseudopregnancy is common which could provide a model system for neuroendocrine studies, but there are ethical issues related to the use of dogs that precludes using female dogs as a model for catamenial epilepsy. Thus, the rodent model is preferable to the canine model when studying perimenstrual seizures.

## Discovery of an Extrasynaptic Mechanism for Targeted Therapy of Catamenial Epielpsy

Neuronal homeostatic systems may regulate the net impact of neuroendocrine factors on neuronal excitability. The GABA-A receptor is a pivotal chloride ionophore for fast inhibitory transmission in the limbic regions relevant to epilepsy pathology. Synaptic γ2-GABA-A receptors mediate the phasic inhibitory currents in response to vesicular release of GABA. They are sensitive to benzodiazepines and neurosteroids. Extrasynaptic δ-GABA-A receptors mediate the tonic inhibitory currents in response to extracellular GABA and play a key role in network excitability (Brickley and Mody, 2012). These extrasynaptic receptors are extremely insensitive to benzodiazepines and highly sensitive to neurosteroids (Carver and Reddy, 2013). Hence, GABA-A receptor plasticity could have an important part to play in the pathophysiology of catamenial epilepsy (Reddy, 2009, 2013a). In 2000, we unexpectedly discovered that neurosteroids, including AP and synthetic analogs such as ganaxolone, showed enhanced anticonvulsant activity in the catamenial epilepsy model (Reddy and Rogawski, 2000b, 2001). Although the model was created based on the NSW paradigm, the molecular mechanism behind neurosteroid’s seizure protection in the catamenial model remained poorly understood. This seminal discovery led us to propose a neurosteroid replacement therapy for prevention of catamenial seizures (Reddy and Rogawski, 2009; Reddy, 2013a). Subsequently we conducted a large array of studies during the past decade to investigate such molecular mechanisms, including progesterone receptors, Egr-3 pathway, and GABA receptor subunit plasticity (Reddy, 2004, 2009, 2013a,b, 2014; Reddy et al., 2004, 2010, 2012; Reddy and Zeng, 2007; Gangisetty and Reddy, 2010; Reddy and Mohan, 2011; Reddy and Ramanathan, 2012). Our most recent studies have ultimately culminated in the discovery of an extrasynaptic mechanism of catamenial epilepsy, one underlying the neurosteroid sensitivity in NSW paradigms (Reddy et al., 2012; Wu et al., 2013; Carver et al., 2014).

**FIGURE 4:**
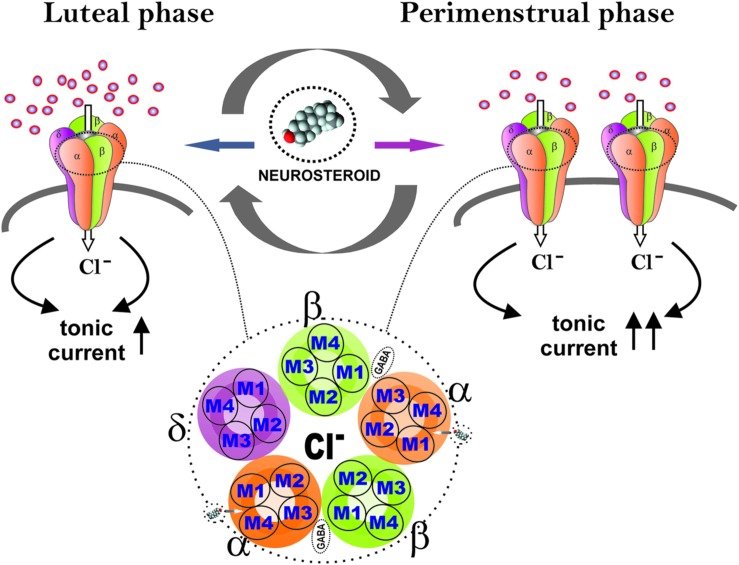
**Extrasynaptic GABA-A receptor plasticity during the ovarian cycle: A perimenstrual delta force mechanism for neurosteroid sensitivity.** During the luteal phase, extrasynaptic δGABA-A receptors in the dentate gyrus contribute to tonic inhibitory currents to relatively elevate the levels of progesterone-derived neurosteroids. At perimenstrual period, neurosteroid levels decline abruptly creating a state of withdrawal. This may trigger the neuronal homeostatic response to increase the δGABA-A receptor abundance to respond to very low levels of neurosteroids, in order to restore tonic currents during the transient withdrawal period.

The application of multidisciplinary approaches has enabled us to uncover the unique plasticity of extrasynaptic δ GABA-A receptors in the dentate gyrus in a mouse model of perimenstrual catamenial epilepsy (Carver et al., 2014). The hippocampal δGABA-A receptors experience plasticity during the menstrual cycle, pregnancy, and parturition (Maguire et al., 2005; Maguire and Mody, 2007; Reddy, 2009; Wu et al., 2013). Hence, we utilized a mouse model of exogenous progesterone/NSW to characterize the GABA-A receptor subunit expression profile in the hippocampus (Gangisetty and Reddy, 2010). We additionally expanded our studies to a mouse gonadotropin/NSW model of catamenial epilepsy (Reddy et al., 2012). In both models, we found a striking increase in the GABA-A receptor δ-subunit, a common partner of α4 in extrasynaptic GABA-A receptors in the dentate gyrus. It is likely that extrasynaptic δGABA-A receptors may endure cyclical plasticity in response to natural fluctuation of hormones during the ovarian cycle and hormone withdrawal period (**Figure 4**). This notion is also supported by the ovarian cycle-related plasticity and function of δGABA-A receptors in the dentate gyrus (Wu et al., 2013). Finally, our electrophysiological studies convincingly demonstrated that the increased expression of the δGABA-A receptors is functionally significant for enhanced AP potentiation of tonic currents in the NSW model (Carver et al., 2014). At the same time, we found smaller tonic GABA current shifts in NSW granule cells compared to controls in the absence of added GABA, indicating low extracellular GABA and other relevant changes in NSW hippocampus. Therefore, the catamenial experimental model is a classical illustration that that the pathogenic origin is due to a rapid shift in tonic current balance in the dentate gyrus, rather than a specific range of AP levels. Dentate granule cells are well known as gatekeepers of seizure propagation to the hippocampus, which explains the observed effects of NSW and AP on seizure susceptibility.

In essence, our studies show that perimenstrual up-regulation of extrasynaptic δGABA-A receptors mediates tonic inhibition in the dentate gyrus granule cells and confers enhanced sensitivity to neurosteroids (**Figure 4**). However, such selective overexpression of δGABA-A receptors is not sufficient to suppress the increased seizure susceptibility triggered by NSW in the absence of AP. Yet, this pathologic increase in δGABA-A receptors may act as a vehicle-catalyst (“Trojan horse”) for the exogenous AP to inhibit seizures (Galanopoulou, 2015). Indeed, this special sensitivity to AP that women with perimenstrual epilepsy exhibit is in agreement with the findings from the NIH progesterone trial, in which the responder group was women with C1 catamenial epilepsy (Herzog et al., 2012). Interestingly, these women responders demonstrated significant posttreatment increase in AP levels (Herzog et al., 2014). Therefore, neurosteroid replacement therapy, proposed earlier, is an ideal therapeutic strategy for C1 catamenial epilepsy (Reddy and Rogawski, 2009; Reddy, 2014). Synthetic neurosteroids given as a brief pulse treatment protocol during this perimenstrual period may be effective in preventing or reducing catamenial seizures. Continuing neurosteroid therapy throughout the menstrual cycle at low doses would contribute little anticonvulsant activity, but would allow patients to avoid negative sedative effects if they are prone to experiencing them. Under both strategies, neurosteroids might provide an effective approach for catamenial epilepsy therapy (Reddy, 2013a). Thus, the cyclical plasticity of an extrasynaptic δGABA-A receptors may serve as lead target for neurosteroid therapy of catamenial epilepsy.

## Conclusion and Future Prospects

Catamenial epilepsy is a complex neuroendocrine condition of refractory menstrual seizures (El-Khayat et al., 2008; Tuveri et al., 2008; Herzog et al., 2014). Neurosteroids are involved in the pathophysiology of catamenial epilepsy. Perimenstrual catamenial (C1) epilepsy is thought to be caused in part by the withdrawal of neuroprotective neurosteroids resulting from the decrease of progesterone levels during the menstrual cycle. There are currently no specific treatments for catamenial seizures; therefore, the NSW model of catamenial epilepsy has been utilized to study treatments for catamenial epilepsy. One important observation is that traditional AEDs have lower efficacy to catamenial seizures. This finding is consistent with women with catamenial epilepsy, who do not respond to these drugs. The model therefore represents a form of the pharmacoresistance paradigm. The higher relative expression of benzodiazepine-insensitive GABA-A receptors during NSW probably explains the reduced activity of benzodiazepines. We found that neurosteroids had enhanced activity in the perimenstrual catamenial epilepsy model. Based on our studies in catamenial epilepsy models, we suggest that “neurosteroid replacement” may be an effective approach to prevent seizure exacerbations often seen with around the menstrual cycles of women. A neurosteroid or a neurosteroid-like drug such as ganaxolone could be given in a “pulse” before menstruation and then either continuously administered at a low dose throughout the month at low doses or discontinued, so as to avoid possible sedative side effects (Reddy, 2014). Therefore, neurosteroids might provide an effective approach for catamenial epilepsy therapy.

The molecular mechanisms underlying the enhanced neurosteroid sensitivity have been reported in mouse models. In a recent publication, we reported the unique plasticity of extrasynaptic δGABA-A receptors in the dentate gyrus of the hippocampus using a mouse perimenstrual model (Reddy et al., 2012; Wu et al., 2013; Carver et al., 2014). A delta-force hypothesis was coined to explain this phenomenon (Galanopoulou, 2015). In summary, the proposed mechanism poses that the perimenstrual decline in neurosteroids triggers the selective overexpression of δGABA-A receptors in the dentate gyrus granule cells, but this is not sufficient to suppress the increased seizure susceptibility of NSW mice in the absence of AP (**Figure 4**). Nevertheless, this compensatory increase in δGABA-A receptors may act as a “Trojan horse” for the exogenous neurosteroid to inhibit seizures. Indeed, this special sensitivity to neurosteroids which women with perimenstrual epilepsy exhibit is in agreement with the findings from the NIH progesterone trial, in which the responder group was women with the perimenstrual type catamenial epilepsy (Herzog et al., 2012). Therefore, the extrasynaptic mechanism represents a molecular rationale for neurosteroid therapy of catamenial epilepsy. Synthetic neurosteroids that enhance tonic inhibition are suitable lead candidates for clinical studies (Carver and Reddy, 2016).

## Author Contributions

The author confirms being the sole contributor of this work and approved it for publication.

## Conflict of Interest Statement

The author declares that the research was conducted in the absence of any commercial or financial relationships that could be construed as a potential conflict of interest.
